# Supplementary material for: Assessment of the Potential Skin Application of Plectranthus ecklonii Benth
Source: Pharmaceuticals (Basel). 2020 Jun 10;13(6):120. doi: 10.3390/ph13060120 (PMC7345374; doi:10.3390/ph13060120)
Supplement: Supplementary file 1 [file pharmaceuticals-13-00120-s001.pdf]

pharmaceuticals

Supplementary material

Structure elucidation of Parvifloron D confirmed by NMR:

Table S1. NMR spectra data of Parvifloron D ($CDCl_3$, ^{1}H-NMR, 400 MHz; ^{13}C-NMR, 100 MHz; δ in ppm, J in Hz).

Position	Δ_H	$J_{H,H}$	Δ_c	HSQC
1α	1.74 dd	(1α, 1β) 13.0	38.37	t
1β	3.76 ddd	(1α, 2β) 11.4		
2β	5.59 tt	(1β, 2β) 4.4	67.87	d
3α	1.56 dd	(2β, 3α) 11.4	45.06	t
3β	2.15 ddd	(2β, 3β) 4.4		
4	--	(3α, 3β) 12.5	38.58	s
5	--	(6, 7) 6.9	164.84	s
6	6.41 d	(15, 16 (17)) 6.8	118.69	d
	--	(14, 15) 0.8		
7	6.79 d	(1β, 3β) 2.4	139.13	d
8	--	(2′, 3′, 5′, 6′) 8.9	127.45	
9	--		127.17	s
10	--		43.91	s
11	--		146.40	s
12	--		178.24	s
13	--		141.61	s
14	6.96 d		133.57	s
15	3.15 sept of d		26.52	d
Me-16*	1.18 d		21.84	d
Me-17*	1.16 d		21.63	q
Me-18	1.29 s		33.03	q
Me-19	1.42 s		30.58	q
Me-20	1.64 s		25.52	s
1′	--		122.43	d
2′,6′	7.93 d		131.89	d
3′,5′	6.88 d		115.23	s
4′	--		160.58	s
7′	--		166.18	
4′-OH	~7.70 br			
11-OH	~7.20 br			

*Interchangeable assignments, ~ not assigned
